# Exploring associations between nutritional intake and cognitive performance in Spanish older adults

**DOI:** 10.3389/fnut.2025.1680012

**Published:** 2025-11-27

**Authors:** Teresa Lopez de Coca, Lourdes Bosch, Victoria Villagrasa, Jakob Norgren, Sara Garcia-Ptacek, Lucrecia Moreno

**Affiliations:** 1 Cátedra DeCo MICOF-CEU UCH, Universidad Cardenal Herrera-CEU, CEU Universities, Valencia, Spain; 2 Department of Pharmacy, Universidad Cardenal Herrera-CEU, CEU Universities, Valencia, Spain; 3 Division of Clinical Geriatrics, Department of Neurobiology, Care Sciences and Society, Karolinska Institutet, Stockholm, Sweden; 4 Aging and Inflammation Theme, Karolinska University Hospital, Stockholm, Sweden

**Keywords:** aging, cognitive health, dietary habits, memory, nutrients

## Abstract

**Background:**

As populations age globally, identifying modifiable dietary factors to support cognitive health has become a pressing research and public health priority. While specific nutrients have been linked to cognitive outcomes, few studies have explored the associations between habitual food group consumption on specific cognitive domains using standardized screening tools.

**Methods:**

To investigate associations between dietary habits, relate to distinct cognitive domains in older adults by analyzing the food consumption in relation to memory, verbal fluency, and global mental status. Spearman correlation coefficients were employed to evaluate associations.

**Results:**

Several nutrient-dense foods, such as nuts, extra virgin olive oil (EVOO), dark chocolate, and eggs, were linked to improved memory and verbal fluency. Conversely, higher intake of dairy products, refined grains, sugary products and red meat correlated with lower cognitive performance, particularly in verbal fluency. A higher proportion of saturated and trans fats, and proteins was inversely associated with cognition, while total fat and monounsaturated fatty acids (MUFA) intake showed positive associations, consistent with the protective role of EVOO.

**Conclusion:**

This study identifies specific, commonly consumed foods that correlate with cognitive function in aging adults. Given the cross-sectional design, associations should not be interpreted as causal, but they underscore the potential of food-based dietary strategies, rather than nutrients specific interventions, to preserve cognitive health and potentially delay age-related cognitive decline.

## 1 Introduction

Dementia is a growing global health challenge. According to the 2024 Lancet Commission on Dementia Prevention, Intervention, and Care, up to 45% of dementia cases may be preventable by addressing 14 modifiable risk factors. While the report does not single out specific foods, several of these factors are diet-related, including obesity (1%), hypertension (2%), type 2 diabetes (2%), high LDL cholesterol (7%), and excessive alcohol intake (1%) (1). Taken together, these figures illustrate that nutrition and lifestyle play a substantial role in dementia prevention, although their effects are overlapping and should not be interpreted as additive. Table 1 presents the 14 modifiable risk factors proposed by the 2024 Lancet Commission

**TABLE 1:** Population attributable fraction of potentially modifiable risk factors for dementia [(1)]

Early life	Midlife	Late life
Less education (5%)	Hearing loss (7%)	Social isolation (5%)
	High LDL cholesterol (7%)	Air pollution (3%)
	Depression (3%)	Visual loss (2%)
	Traumatic brain injury (3%)	
	Physical inactivity (2%)	
	Diabetes (2%)	
	Smoking (2%)	
	Hypertension (2%)	
	Obesity (1%)	
	Excessive alcohol (1%)	

Dietary patterns play a central role in maintaining brain health and reducing cognitive decline. Current evidence supports the adoption of healthy dietary patterns, particularly the Mediterranean, MIND (Mediterranean-DASH Intervention for Neurodegenerative Delay), and Nordic diets, as effective strategies for cognitive preservation (2). These diets emphasize plant-based, nutrient-rich foods and have been associated with slower rates of cognitive aging. However, practical challenges such as food insecurity, cultural variability, and limited nutrition education among healthcare providers hinder the translation of evidence into practice.

Emerging research has highlighted the role of chronic systemic inflammation as a key mechanism linking poor dietary quality with cognitive impairment and neurodegeneration. Anti-inflammatory diets have therefore been proposed as a promising strategy to mitigate this risk. Recent studies indicate that consistent adherence to such diets may protect against cognitive decline, physical impairments, depressive symptoms, and multiple chronic conditions. Accordingly, a well-designed anti-inflammatory dietary approach may represent a viable, non-pharmacological intervention to prevent age-related cognitive deterioration in older adults (3). At the biological level, nutrients influence synaptic plasticity and neuronal signaling pathways. Brain-derived neurotrophic factor (BDNF), a critical mediator of neurogenesis and synaptic growth, is modulated by specific dietary components. Omega-3 fatty acids, flavonoids, and B-vitamins, among others, have shown potential in preserving cognitive function and delaying the onset of neurodegenerative processes (4).

Certain food groups have been individually associated with cognitive outcomes. For instance, high intake of dairy products, especially those rich in saturated fats, has been linked to poorer cognitive performance, potentially via pro-inflammatory and vascular mechanisms (5). In contrast, foods rich in polyphenols, such as berries, tea, and EVOO, may exert neuroprotective effects through both antioxidant and anti-inflammatory pathways (6–8). Blue fish, a major source of omega-3 fatty acids and nuts, which provide vitamin E, unsaturated fats, flavonoids and selenium, may contribute to neuroprotection. In addition, nuts contain arginine, a semi-essential amino acid and precursor of nitric oxide, which plays a role in vascular health, and have also been associated with improved memory and executive function (9–11). Conversely, diets high in refined grains, sugary products, and red or processed meats may contribute to cognitive decline via insulin resistance, oxidative stress, and cerebrovascular impairment (12–14).

For that reason, public health strategies promoting anti-inflammatory and nutrient-rich dietary patterns, along with physical activity, smoking cessation, and social engagement, deserve further investigation as potential tools to reduce the global burden of dementia. Nonetheless, further research is needed to determine which specific dietary factors most strongly support cognitive function.

This study aims to explore the relationship between dietary patterns and cognitive performance. Specifically, it examines the intake of selected food groups and macronutrient parameters based on their pro- or anti-inflammatory potential, as defined by current evidence on anti-inflammatory dietary models (15). These categories include both foods considered beneficial, and those identified as potentially harmful, such as refined grains, alcohol, or added sugars. Rather than evaluating adherence to an anti-inflammatory dietary pattern *per se*, this study utilizes the food group classification proposed by the AnMED model. This framework, conceptualized as a restrictive variant of the Mediterranean diet, similar to the MIND diet but with a stricter exclusion of pro-inflammatory components, was used to analyze individual food groups in relation to cognitive test outcomes. Cognitive performance was assessed using three standardized tools that target distinct cognitive domains. Accordingly, the study tests the hypothesis that intake levels of food groups classified within the AnMED framework are associated with variations in cognitive test performance, without assuming *a priori* the direction of these associations.

## 2 Materials and methods

### 2.1 Study design

This is a cross-sectional study. Participants were invited to participate through community pharmacies and healthcare centers in the Valencian Community (Spain) from April to October 2024.

The inclusion criteria were to be at least 50 years old and willing and able to provide written informed consent. Exclusion criteria included those individuals with any diagnosis of dementia, intellectual disability, and/or those experiencing severe sensory deficits (such as blindness or deafness) or physical disability that impaired the ability to participate in the interview.

### 2.2 Participants and data collection

Data collection included a comprehensive personal interview lasting approximately 90 min, allowing for detailed and reliable information. Sociodemographic and anthropometric data were collected, including age, sex, education and body mass index (BMI).

#### 2.2.1 Nutritional data collection

Dietary data were collected through a personal interview, which also included demographic variables such as age and gender. All information was anonymized and compiled into a secure database. To assess nutritional intake, the Food Frequency Questionnaire (FFQ) developed by the PREDIMED study group was used (16). This instrument recorded the frequency of consumption of food portions or whole pieces over the preceding month. Portion sizes were derived directly from the standardized weights provided in the PREDIMED questionnaire; no *ad hoc* conversions were applied. Frequencies were then converted into estimated daily intake values (in grams) based on these predefined portion weights. Nutrient content and bioactive compound composition were derived from the Souci-Fachmann-Kraut Food Composition and Nutrition Tables (9th edition) (17).

The reported foods were subsequently grouped into categories according to the classification proposed by Sala-Climent et al. (15), which defines an anti-inflammatory dietary model based on the inflammatory potential of foods. This classification includes both beneficial (healthy or anti-inflammatory foods categories) and potentially harmful (pro-inflammatory) food groups. Importantly, the global scoring system of the AnMED index was not applied. Detailed information on these food categories is provided in Supplementary material 1.

For each participant, energy contributions from macronutrients were calculated using the Atwater conversion factors (4 kcal/g for carbohydrates and protein, and 9 kcal/g for fat), Nutrient composition information was derived from the Souci-Fachmann-Kraut Food Composition and Nutrition Tables (17). We additionally derived the breakdown of MUFA, polyunsaturated fatty acids (PUFA), trans fats, and saturated fatty acids (SFA), along with the following dietary ratios: carbohydrate-to-fat (rCarb/Fat), saturated fat-to-total fat (rSFA/Fat), and saturated fat-to-unsaturated fat [rSFA/(MUFA + PUFA)]. These variables were used in subsequent statistical analyses.

#### 2.2.2 Cognitive performance

Cognitive testing was evaluated using three validated cognitive screening tools: the Memory Impairment Screen (MIS) (18), the Semantic Verbal Fluency test (SVF) (19), and the Short Portable Mental State Questionnaire (SPMSQ) (20). The use of multiple tests with distinct sensitivity and specificity profiles enhances diagnostic accuracy.

The MIS is a brief test designed to assess memory function through four items that evaluate free and facilitated recall. It uses controlled learning to optimize encoding and recall. The test is scored on an 8-point scale, with a cut-off point of ≤ 4 indicating potential memory impairment. In the Spanish population, it has demonstrated high diagnostic accuracy, with a sensitivity of 80 and a specificity of 96% (18). The MIS is particularly effective in identifying early deficits related to Alzheimer’s disease and other forms of cognitive impairment, making it a reliable screening tool for episodic memory function.

The SVF test evaluates lexical access and semantic memory by asking individuals to name as many items as possible from a given semantic category (e.g., animals) within 1 min. A score of fewer than 10 words is generally considered indicative of cognitive impairment. The test is simple and time-efficient, and it has shown a sensitivity of 74 and a specificity of 80% in detecting cognitive decline (19). The SVF is especially useful for identifying impairments in the temporal and frontal lobes, where progressive semantic memory loss is commonly observed in amnestic mild cognitive impairment.

The SPMSQ test is a screening instrument that assesses global cognitive status. It includes 10 questions covering domains such as short- and long-term memory, orientation, general knowledge, and basic arithmetic abilities. The test is scored by counting the number of errors, with a maximum score of 10 indicating the highest number of mistakes. The original scoring system classifies cognitive performance as follows: 0–2 errors indicate intact cognitive function; 3–4 errors suggest mild cognitive impairment; and 5 or more errors indicate severe impairment. The Spanish adaptation of the SPMSQ has demonstrated a sensitivity of 85.7% and a specificity of 79.3% (20). Due to its brevity and ease administration, the SPMSQ is suitable for use in various clinical and community settings to evaluate overall cognitive function.

However, because the SPMSQ scores are inversely related to cognitive performance, where higher scores indicate worse cognition, it was necessary to reverse its scoring for this study. In contrast, the other two cognitive tests used, the MIS and SVF tests, assign higher scores to better cognitive function. To enable clearer interpretation and facilitate cross-comparison between tests, the SPMSQ scores were inverted (i.e., 0 became 10, 1 became 9, and so on), so that in all three assessments, higher scores consistently represented better cognitive performance. This harmonization improves the readability of the results and the consistency of graphical and statistical outputs, ensuring that cognitive scores are interpreted in the same direction across all analyses.

### 2.3 Statistical inference

All dietary intake data were compiled in an Excel spreadsheet and analyzed using RStudio (version 4.4.2). Macronutrient energy contributions and specific nutrient ratios were computed to support statistical modeling. As a first step, the population was categorized based on cognitive status, and differences in daily intake values (in grams) between groups were evaluated using the Mann–Whitney U test with *p*-value adjustment (False Discovery rate (FDR) method) to control for multiple comparisons. To move beyond unadjusted comparisons, the primary analytical framework relied on multivariable regression models. For each cognitive test, the score was regressed on the nutritional exposure and adjusted *a priori* for age and educational attainment. These two covariates were selected as the minimal sufficient adjustment set, based on prior evidence and causal reasoning supported by a directed acyclic graph. Age and education are well-established determinants of cognitive performance and plausible common causes of both dietary patterns and cognition. This adjustment strategy was designed to block major confounding paths while avoiding overfitting and collider bias.

Sex was considered as a potential confounder or effect modifier but was not included in the primary models. Instead, three pre-specified sensitivity checks were performed for each exposure–outcome pair: (1) adding sex as a covariate, (2) testing exposure × sex interactions, and (3) conducting sex-stratified models. These complementary analyses are reported in the Supplementary material 2.

Following this, Spearman’s rank correlation coefficients (ρ) were used to explore correlations between dietary exposures and cognitive outcomes, based on the intake of each food group measured in grams per day. All *p*-values were corrected for multiple testing using FDR within each cognitive outcome, and we report both raw and *q*-values to allow assessment of robustness. All analyses were performed in R, employing a set of well-established libraries: *dplyr* and *tidyr* for data wrangling, *ggplot2* and *ggcorrplot* for data visualization, *Hmisc* for correlation analysis (via the rcorr function), and the tidyverse suite for efficient and coherent data management. This integrated approach ensured analytical transparency, reproducibility, and adherence to robust statistical practices.

### 2.4 Sample size

The sample size calculation was performed using the pwr package in RStudio, targeting the detection of a minimum Spearman correlation coefficient of 0.14 between dietary variables and cognitive test scores. A significance level (α) of 0.05 and a statistical power (1 −β) were specified for a two-sided test. This calculation yielded a minimum required sample size of 398 participants. Given the observed correlation strengths reported in similar studies and within this investigation, this sample size was considered sufficient to detect meaningful associations between nutritional intake and cognitive performance.

### 2.5 Ethical approval and data protection

The studies involving human participants were reviewed and approved by the Institutional Review Board (IRB) at the CEU Cardenal Herrera University (CEEI24/533, date of approval: 25 Abril 2024). The participants provided their written informed consent to engage in this study.

Information processing guarantees both the protection of the data and their security. This data was treated confidentially and lawfully and were used for the purpose for which the participant had been informed. Thus, this work complied with the European General Data Protection Regulation and Organic Law 3/2018 on the Protection of Personal Data and the Guarantee of Digital Rights. The study compiled with the basic principles of the Declaration of Helsinki: respect for the individual (Article 8) and recognition of their right to self-determination and their right to make informed decisions (informed consent, contained in Articles 20, 21, and 22), including participation in research, both at its beginning and throughout the study.

## 3 Results

A total of 531 participants were included in the analysis of food consumption frequency. The mean age of the sample was 69.32 ± 11.73 years, and the majority were women (87.19%). The average BMI was 26.85 ± 4.67 kg/m^2^. Mean daily energy intake was 1674.73 ± 476.87 kcal, with an average macronutrient distribution of 52.86% from fats, 13.02% from proteins, and 34.11% from carbohydrates. Regarding cognitive performance, 19.21% of participants screened positive for cognitive impairment (CI) in at least one of the tests.

**FIGURE 1:**
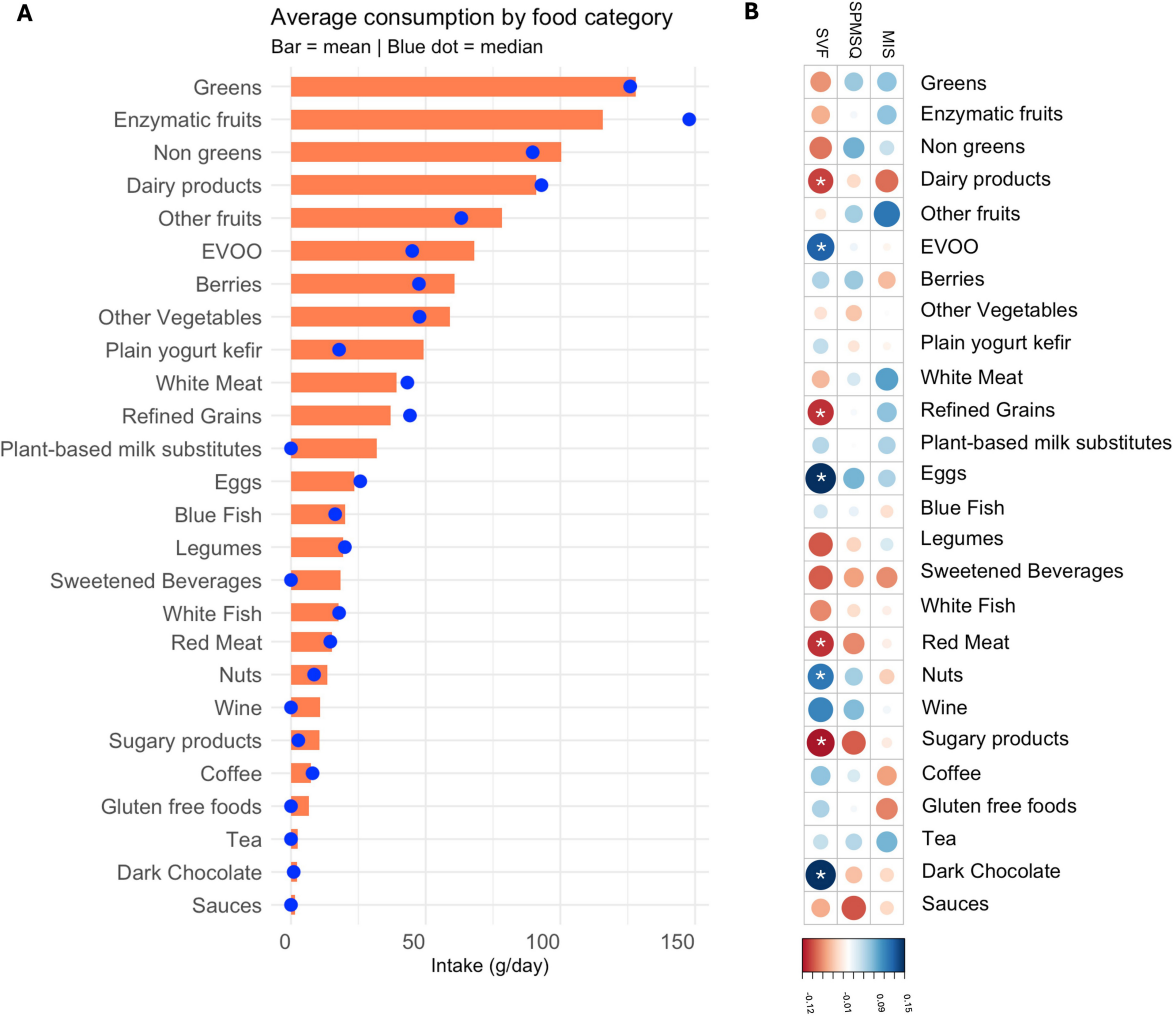
**(A)** Distribution of food consumption (mean vs. median). **(B)** Spearman correlation coefficients between the amount of food consumed (g/day) and cognitive performance on three tests: SVF, SPMSQ, and MIS. Cooler colors indicate positive associations, and warmer tones indicate negative correlations. Significant codes: adjust *p< 0.05.

As a preliminary step, the distribution of intake across 27 food categories was explored to assess the underlying structure of dietary data. Figure 1A displays both the mean and median intake for each category, in terms of g/day revealing notable discrepancies in several items, particularly those with skewed or sparse consumption patterns. These asymmetries reflect the non-normal and heterogeneous nature of dietary data in free-living populations. Considering this, multivariable regression models adjusted *a priori* for age and educational levels were adopted as the primary analytical framework, with exploratory Spearman’s rank correlations. Spearman’s rank correlation was selected as the most appropriate method to examine the relationship between food intake and cognitive scores, with correlations adjusted for participants’ age and educational levels and *p*-values corrected for multiple comparisons using FDR method. Its robustness to outliers and lack of distributional assumptions make it well suited for identifying monotonic associations in this context. This approach enabled a more nuanced, continuous investigation of dietary influences on cognition, beyond binary group comparisons or strictly linear models.

Figure 1B presents the Spearman correlation coefficients between the quantities of consumption of the different food groups and cognitive performance as measured by the MIS, SPMSQ, and SVF tests. Since all scores have been aligned so that higher values consistently reflect better cognitive performance, the direction and strength of these associations provide insight into the cognitive relevance of dietary patterns.

After adjustment for age and educational level, only associations with SVF remained significant. Detrimental correlations were observed for sugary products (ρ: −0.122), red meat (ρ: −0.108), refined grains (ρ: −0.107) and dairy products (ρ: −0.100). Positive associations with SVF were observed for eggs (ρ: 0.148), dark chocolate (ρ: 0.145), EVOO (ρ: 0.119), and nuts (ρ: 0.106). Data can be found in Table 2. No significant associations were found for MIS or SPMSQ.

**TABLE 2:** Spearman correlation coefficients between dietary food frequency and cognitive test scores.

	MIS			SPMSQ			SVF		
Foods categories	ρ	*p*	*q*	ρ	*p*	*Q*	ρ	*p*	*q*
Greens	0.058	0.180	0.477	0.054	0.217	0.614	−0.065	0.133	0.246
Enzymatic fruits	0.058	0.183	0.477	0.007	0.868	0.933	−0.053	0.227	0.347
Non-greens	0.033	0.455	0.786	0.07	0.110	0.586	−0.078	0.071	0.154
Dairy products	−0.082	0.060	0.477	−0.028	0.522	0.804	−0.100	0.002	0.014*
Other fruits	0.107	0.014	0.357	0.049	0.260	0.614	−0.017	0.696	0.696
EVOO	−0.008	0.854	0.888	0.009	0.838	0.933	0.119	0.006	0.027*
Berries	−0.046	0.287	0.574	0.054	0.214	0.614	0.046	0.295	0.384
Other vegetables	0.003	0.948	0.948	−0.040	0.360	0.668	−0.023	0.592	0.615
Plain yogurt or kefir	−0.009	0.841	0.888	−0.020	0.653	0.849	0.036	0.415	0.482
White meat	0.081	0.064	0.477	0.026	0.553	0.804	−0.048	0.269	0.384
Refined grains	0.06	0.166	0.477	0.006	0.897	0.933	−0.107	0.001	0.012*
Plant-based milk substitutes	0.047	0.285	0.574	−0.002	0.964	0.964	0.042	0.331	0.410
EGGS	0.046	0.287	0.574	0.069	0.113	0.586	0.148	0.001	0.011*
Blue fish	−0.024	0.575	0.787	0.014	0.748	0.926	0.029	0.505	0.547
Legumes	0.025	0.569	0.787	−0.032	0.461	0.799	−0.091	0.036	0.092
Sweetened beverages	−0.068	0.116	0.477	−0.060	0.166	0.614	−0.090	0.039	0.092
White fish	−0.012	0.777	0.888	−0.025	0.559	0.804	−0.070	0.110	0.220
Red Meat	−0.013	0.764	0.888	−0.071	0.104	0.586	−0.108	0.013	0.047*
Nuts	−0.034	0.430	0.786	0.049	0.256	0.614	0.106	0.015	0.047*
Wine	0.009	0.840	0.888	0.064	0.14	0.607	0.097	0.026	0.076
Sugary products	−0.016	0.716	0.888	−0.089	0.04	0.524	−0.122	0.005	0.025*
Coffee	−0.060	0.170	0.477	0.024	0.587	0.804	0.059	0.175	0.304
Gluten free foods	−0.073	0.093	0.477	0.006	0.892	0.933	0.047	0.284	0.384
Tea	0.068	0.117	0.477	0.042	0.335	0.668	0.035	0.426	0.482
Dark chocolate	−0.029	0.501	0.786	−0.043	0.321	0.668	0.145	0.001	0.011*
Sauces	−0.028	0.514	0.786	−0.093	0.032	0.524	−0.053	0.219	0.347

*Values shown are correlation coefficients (ρ), nominal *p*-values (p), and FDR-adjusted *p*-values (q). MIS: Memory Impairment Screen. SPMSQ: Short Portable Mental State Questionnaire. SVF: Semantic Verbal Fluency. *p < 0.05.

### 3.1 Energy intakes and ratios

Macronutrient intake was assessed for all participants, and Spearman correlation analyses were conducted to explore the relationships between dietary variables and cognitive/functional outcomes.

**FIGURE 2:**
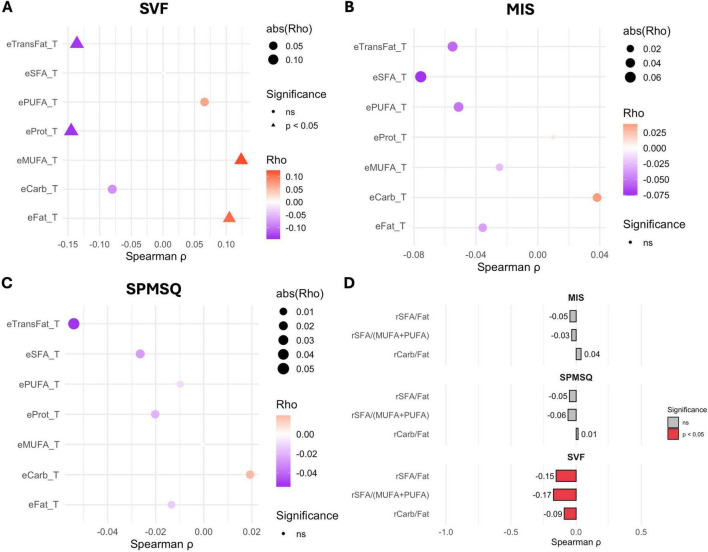
Spearman correlation between energy intake from macronutrients and performance on cognitive tests. **(A)** Semantic Verbal Fluency (SVF). **(B)** Memory Impairment Screen (MIS). **(C)** Short Portable Mental Status Questionnaire (SPMSQ). **(D)** Correlation between ratios on cognitive test. Significant correlations are indicated by triangles (adjust *p* < 0.05); the direction and strength are represented by the color and size of the points, respectively.

After adjusting for age and educational level, only the associations with SVF remained significant (Figure 2). Specifically, negative correlations were observed with trans-fat intake (ρ: −0.136) and protein intake (ρ: −0.145), indicating worse verbal fluency performance with higher consumption of these nutrients. In addition, inverse correlations were found with the SFA/Fat ratio (ρ: −0.154) and the SFA/(MUFA + PUFA) ratio (ρ: −0.175), reinforcing the detrimental role of dietary saturated fats. Conversely, positive associations emerged for total fat (ρ: −0.105) and MUFA intake (ρ: −0.124). The correlation data, *p*-values, and their adjustments can be seen in Table 3. No significant associations persisted for MIS or SPMSQ.

**TABLE 3:** Spearman correlation coefficients between dietary macronutrients intake and cognitive test scores.

	MIS			SPMSQ			SVF		
Variable	ρ	p	q	ρ	*p*	q	ρ	p	q
eTransFat_T	−0.055	0.206	0.542	−0.054	0.214	0.783	−0.136	0.002	0.004*
eSFA_T	−0.076	0.082	0.542	−0.026	0.544	0.914	0.001	0.98	0.98
eProt_T	0.01	0.822	0.822	−0.02	0.643	0.914	−0.145	0.001	0.003*
eFat_T	−0.036	0.414	0.542	−0.013	0.758	0.914	0.105	0.016	0.026*
eMUFA_T	−0.025	0.57	0.633	0	0.992	0.992	0.124	0.004	0.009*
eCarb_T	0.038	0.381	0.542	0.019	0.658	0.914	−0.08	0.066	0.082
ePUFA_T	−0.051	0.24	0.542	−0.01	0.823	0.914	0.066	0.131	0.146
rCarb/Fat	0.038	0.387	0.542	0.015	0.731	0.914	−0.092	0.034	0.049*
rSFA/Fat	−0.048	0.272	0.542	−0.052	0.235	0.783	−0.154	0	0.002*
r SFA/(MUFA + PUFA)	−0.034	0.434	0.542	−0.062	0.152	0.783	−0.175	0	0.001*

*Values shown are correlation coefficients (ρ), nominal *p*-values (p), and FDR-adjusted *p*-values (q). MIS, Memory Impairment Screen; SPMSQ, Short Portable Mental State Questionnaire; SVF, Semantic Verbal Fluency. *p < 0.05.

## 4 Discussion

This study is made to explore associations between specific dietary components and cognitive performance. To this end, MIS, SPMSQ, and SVF assess distinct domains of cognitive functioning. Higher scores on the MIS and SVF indicate better cognitive performance, whereas lower scores on the SPMSQ reflect fewer errors and, therefore, better global cognitive status. It was necessary to reverse its scoring for this tool to enable clearer interpretation with the other tools. Through a correlational approach, this analysis aimed to identify dietary patterns potentially linked to either cognitive preservation or decline, providing insights into how certain food intake may influence brain health.

The MIS, which evaluates episodic memory and relies mainly on the hippocampus and medial temporal lobe, did not retain any significant dietary associations after adjustment for age and education, suggesting a more limited sensitivity of this test to nutritional influences in this cohort. The SVF test, which assesses verbal fluency and semantic memory, is functionally related to the temporal and frontal lobes, retained multiple associations after adjustment, highlighting its potential role as the most diet-sensitive test among those analyzed. It showed positive associations with various foods groups: nuts (ρ: 0.106; *p*-value: 0.049), dark chocolate (ρ: 0.145; *p*-value: 0.011), EVOO (ρ: 0.119; *p*-value: 0.034), and eggs (ρ: 0.148, *p*-value: 0.011), These results support the idea that diets rich in healthy fats (e.g., omega-3), antioxidants, choline, and flavonoids could promote better functional connectivity in frontotemporal regions. Other negative associations with SVF were observed for sugary products (ρ: −0.112, *p*-value: 0.033), dairy products (ρ: −0.011; *p*-value: 0.044), refined grains (ρ: −0.013; *p*-value: 0.047) and red meat (ρ: −0.108, *p*-value: 0.049) reinforcing the detrimental role of high-fat/sugar foods in verbal and lexical domains.

The SPMSQ, which assesses global cognitive status by measuring errors in memory, orientation, arithmetic, and general knowledge, did not show significant associations with dietary intake, reflecting its broader but less domain-specific sensitivity compared to SVF.

After adjustment for age and educational level, all three nutrient ratios became significantly associated with SVF. Inverse correlations were observed for the carbohydrate-to-fat ratio (ρ: −0.092, p-value: 0.049), contrasting with a previous study that reported a modest negative association between this ratio and cognitive function, while observing an inverted U-shaped relationship for the saturated-to-total fat ratio. The contrasting findings for the carbohydrate-to-fat ratio could potentially be explained by differing prevalence of APOE genotypes in northern versus southern Europe (21), the SFA/Fat ratio (ρ: −0.154, *p*-value: 0.002), and the SFA/(MUFA + PUFA) ratio (ρ: −0.175, *p*-value: 0.001), indicating that a higher relative contribution of saturated fats and carbohydrates was linked to poorer verbal fluency. In addition, negative associations were found with protein intake (ρ:−0.136, *p*-value: 0.003) and trans fats (ρ:−0.136, *p*-value: 0.004), whereas positive associations emerged for total fat (ρ: 0.105, *p*-value: 0.026) and MUFA intake (ρ: 0.124, *p*-value: 0.009). The relationship between the consumption of saturated and trans fats and cognitive decline has been extensively studied (22, 23). Multiple studies have found a positive association between high intake of saturated fats and an increased risk of cognitive decline, including Alzheimer’s disease (AD) and mild cognitive impairment (MCI). For instance, higher saturated fat intake was linked to a greater risk of AD in 3 out of 4 studies and cognitive decline in 2 out of 4 studies (24). Another study reported that the highest category of saturated fat intake was associated with a 40% increased risk of cognitive impairment and an 87% increased risk of AD (25). This combination of fat may exacerbate cognitive impairment through mechanisms involving oxidative stress, inflammation, and insulin resistance (23).

Common nuts have nutrients and bio-actives including monounsaturated and poly-unsaturated fatty acids, high-quality protein, fiber, non-sodium minerals, tocopherols, phytosterols, and antioxidant phenolics (26). It is well known that the daily consumption of nuts may play a protective role in the prevention of dementia. Specifically, consumption of up to 1 handful of 30 g/day of unsalted nuts were associated with the greatest protective benefits. Higher consumption of nuts has been associated with a lower prevalence of cognitive impairment in older adults (27, 28). Consumption of a trail mix has been shown to improve visuospatial and verbal memory and cerebral vascular function, which may underlie improvements in cognitive performance (29).

EVOO consumption has been increasingly recognized for its neuroprotective properties, attributed to its high content of MUFA and bioactive polyphenols. Notably, the positive association observed between MUFA intake and verbal fluency in our analyses aligns with the beneficial role of EVOO, where MUFA is the predominant fatty acid. Clinical evidence supports its role in maintaining brain health: in a randomized controlled trial, Kaddoumi et al. (30) demonstrated that EVOO improved brain connectivity and reduced blood–brain barrier permeability in individuals with mild cognitive impairment, suggesting a mechanistic pathway through which olive oil biophenols may protect against neurodegeneration. Long-term epidemiological data (31), further reinforce these effects; this study found that consuming more than 7 g/day of olive oil was associated with a 28% lower risk of dementia-related mortality, independent of overall diet quality. These findings converge to indicate that regular EVOO consumption, through its MUFA-rich and polyphenol content, may contribute to preserving cognitive function and reducing the risk of progression to dementia.

Egg consumption has been associated with neuroprotective effects, supported by the presence of bioactive compounds such as choline, lutein, zeaxanthin, tryptophan, and docosahexaenoic acid (32, 33). These nutrients are involved in neurotransmission, antioxidative defense, and synaptic integrity, and may help attenuate neuroinflammatory responses (33). Epidemiological evidence indicates that moderate egg intake is linked to enhanced cognitive function and a reduced risk of dementia, with protective effects largely mediated by dietary choline (34). Furthermore, regular egg consumption has been associated with less decline in verbal fluency (35), reinforcing our observation of its positive association with SVF performance.

Dark chocolate consumption has been associated with cognitive benefits, mainly attributed to its high content of flavonoids, which can modulate synaptic plasticity and cerebral blood flow (36). Evidence suggests that regular intake is linked to improved memory performance and reduced risk of cognitive decline in older adults (37). In addition, protective effects on mental fatigue and cognitive function have been observed in middle-aged populations (38). These findings support the role of cocoa-derived polyphenols in promoting brain health and are consistent with our observation of a positive association between dark chocolate intake and SVF scores.

Taken together, these findings support the hypothesis that dietary components exert differential effects across different cognitive domains. Foods rich in polyphenols, antioxidants, and unsaturated fats appear particularly beneficial for verbal fluency and semantic memory, likely due to their neuroprotective, anti-inflammatory, and vascular-modulating properties. In contrast, high intake of sugars, trans and saturated fats, appears to disproportionately impair lexical access. Understanding these domain-specific dietary associations could inform targeted nutritional strategies to help preserve cognitive health in aging populations.

### 4.1 Strengths and limitations

First, data collection was based on a comprehensive face-to-face interview lasting approximately 90 min, ensuring the acquisition of detailed and reliable information on dietary intake and lifestyle. Second, cognitive function was assessed using three complementary tests (MIS, SPMSQ, and SVF), each capturing different cognitive domains with varying degrees of sensitivity and specificity, thereby allowing for a broader and more nuanced evaluation of cognitive performance. Third, the sample consisted of individuals from a Mediterranean population, characterized by relatively homogeneous environmental, cultural, and dietary contexts, which enhances the internal validity of the findings. Finally, one of the key strengths of this study lies in the detailed assessment of the full dietary profile, rather than relying solely on macronutrient percentages or simplified nutrient ratios. This comprehensive nutritional approach enabled the identification of specific dietary components associated with cognitive outcomes, offering greater depth and clinical relevance to the findings.

However, certain limitations should be acknowledged. First, the cross-sectional design precludes causal inferences between dietary components and cognitive performance, consequently, all findings should be interpreted strictly as associations rather than causal effects. Second, although potential confounding variables, such as comorbidities, medication use, and physical activity, were examined at the beginning of the analysis and no significant differences were found across cognitive outcomes, these factors were not included in further statistical adjustments. Residual confounding, including from unmeasured confounders cannot be completely ruled out. Finally, while the sample size was sufficient to detect small-to-moderate associations, the exploratory nature of the study calls for confirmation through longitudinal or interventional research.

## 5 Conclusion

This study reveals small but meaningful associations between specific dietary components and cognitive performance in older adults. Among the protective factors, include foods such as EVOO, nuts, dark chocolate and eggs, which contain polyphenols, choline, unsaturated fats and/or other bioactive compounds to support brain health. Conversely, dairy products, sugary products, refined grains and red meat were negatively associated with cognitive outcomes, suggesting that frequent consumption of these items, often high in saturated fats, sugars, or additives, may contribute to cognitive decline.

Verbal fluency emerged as the cognitive function most influenced by dietary variations, this could be due to its dependence on widespread and integrative neural networks involved in language production, semantic access, and executive control. This highlights the potential vulnerability of verbal fluency to nutritional factors and reinforces the importance of dietary patterns in supporting complex cognitive processes.

These findings support the promotion of brain-healthy dietary patterns as a modifiable factor for maintaining cognitive function in aging populations. Future longitudinal and intervention studies are warranted to confirm these associations and elucidate underlying mechanisms.

## Data Availability

The raw data supporting the conclusions of this article will be made available by the authors, without undue reservation.
