# Supplementary material for: Exploring associations between nutritional intake and cognitive performance in Spanish older adults
Source: Front Nutr. 2025 Nov 27;12:1680012. doi: 10.3389/fnut.2025.1680012 (PMC12695553; doi:10.3389/fnut.2025.1680012)
Supplement: Supplementary file 1 [file Table_1.docx]

Supplementary Material

# Supplementary Table

Examples of foods categories from an anti-inflammatory dietary pattern.

| **Berries** | Strawberries, cherries, plums, raspberries, blueberries |
| --- | --- |
| **Blue fish** | Herring, tuna, salmon, trout, sardines |
| **Coffee** | Coffee |
| **Dairy products** | Whole milk, semi-skimmed milk, skimmed milk, condensed milk, cream, sweetened yogurt, cured or fresh cheese, cream cheese, processed cheese portions, butter. |
| **Dark chocolate** | Dark chocolate with more than 80% cocoa |
| **Eggs** | Egg |
| **Enzymatic fruits** | Orange, grapefruit, mandarins, pineapple, banana, kiwi, grape, raisins, fresh orange juice. |
| **EVOO** | Extra virgin olive oil, virgin olive oil, olive oil |
| **Gluten free foods** | Oat bread, buckwheat bread, brown rice, white rice, corn, potato. |
| **Greens** | Chard, spinach, lettuce, endives, escarole, arugula, green beans. |
| **Legumes** | Lentils, beans, chickpeas, peas. |
| **Non-greens** | Raw tomato, carrot, artichokes, celery, peppers, asparagus, mushrooms. |
| **Nuts** | Almond, walnuts, hazelnuts. |
| **Other fruits** | Apple, pear, peach/nectarine, apricot, melon, watermelon, preserved pear, preserved apricot, preserved peach, dates, dried figs. |
| **Other vegetables** | Cabbage, cauliflower, broccoli, avocado, pumpkin, eggplant, zucchini, cucumber, leek, onion, garlic baked or boiled potatoes, sweet potato, homemade French fries, commercial French fries, olives. |
| **Plant-based milk substitute** | All nut-derivate drinks, soy and oat drinks |
| **Plain yogurt/kefir** | Plain yogurt (without sugar), kefir |
| **Red meat** | Beef, pork, lamb, liver, brains, heart, sweetbread, Serrano ham, cooked ham, bacon, lard, pancetta, hamburger or meatballs. |
| **Refined grains** | Whole wheat bread, wheat bread, rye bread, barley bread, breakfast cereals, pasta, whole grain or fiber cookies, chocolate cookies, croissant, muffins, dinner rolls, donut, cakes, Spanish fried dough pastry, snacks other than potato chips: popcorn, corn puffs, cheese puffs. |
| **Sauces and jam** | Mayonnaise, mustard, ketchup, fried tomato sauce, orange marmalade, peach marmalade, strawberry jam, raspberry jam, and other jam/marmalades that have not specifically been described before. |
| **Sugary products** | Ice cream, Marie cookies, homemade pastries and cakes, chocolate and pralines, cocoa powder, marzipan, sugar, honey. |
| **Sweetened beverages** | Cola, sweetened soft drinks, tonic water, industrial orange juice. |
| **Tea** | Green tea, red tea, white tea, black tea. |
| **White fish** | Cod, hake, sole, mussels, clams, shrimp. |
| **White meat** | Chicken, turkey, rabbit, hare. |
| **Wine** | Red wine, white wine, rose wine. |
