# Supplementary material for: Exploring associations between nutritional intake and cognitive performance in Spanish older adults
Source: Front Nutr. 2025 Nov 27;12:1680012. doi: 10.3389/fnut.2025.1680012 (PMC12695553; doi:10.3389/fnut.2025.1680012)
Supplement: Supplementary file 2 [file Table_2.docx]

Supplementary Material

# 2 Supplementary Table

Directed acyclic graph

| **term** | **estimate** | **SD** | **p-value** | **Outcome** | **Exposure** | **Covars** | **SEX** | **adjust p** |
| --- | --- | --- | --- | --- | --- | --- | --- | --- |
| Blue Fish | 0.006 | 0.003 | 0.048 | SPMSQ | Blue Fish | Age, study levels and sex | All | 0.601 |
| Blue Fish | 0.005 | 0.003 | 0.098 | SPMSQ | Blue Fish | Age and study levels | All | 0.603 |
| Blue Fish | 0.006 | 0.003 | 0.075 | SPMSQ | Blue Fish | Age and study levels | Fem | 0.603 |
| Blue Fish | 0.008 | 0.007 | 0.313 | SPMSQ | Blue Fish | Age and study levels | Masc | 0.754 |
| Blue Fish, masc sex | 0.002 | 0.009 | 0.838 | SPMSQ | Blue Fish, sex | Age and study levels | All | 0.956 |
| Blue Fish | -0.014 | 0.01 | 0.177 | MIS | Blue Fish | Age and study levels | Masc | 0.677 |
| Blue Fish | -0.004 | 0.004 | 0.21 | MIS | Blue Fish | Age and study levels | All | 0.721 |
| Blue Fish | -0.004 | 0.004 | 0.221 | MIS | Blue Fish | Age, study levels and sex | All | 0.721 |
| Blue Fish | -0.003 | 0.004 | 0.419 | MIS | Blue Fish | Age and study levels | Fem | 0.797 |
| Blue Fish, masc sex | -0.011 | 0.012 | 0.355 | MIS | Blue Fish, sex | Age and study levels | All | 0.759 |
| Blue Fish | 0.013 | 0.016 | 0.418 | SVF | Blue Fish | Age and study levels | Fem | 0.797 |
| Blue Fish | 0.007 | 0.015 | 0.615 | SVF | Blue Fish | Age and study levels | All | 0.898 |
| Blue Fish | 0.004 | 0.015 | 0.788 | SVF | Blue Fish | Age, study levels and sex | All | 0.949 |
| Blue Fish, masc sex | -0.08 | 0.048 | 0.097 | SVF | Blue Fish, sex | Age and study levels | All | 0.603 |
| Blue Fish | -0.065 | 0.047 | 0.171 | SVF | Blue Fish | Age and study levels | Masc | 0.677 |
| Dairy products | 0 | 0.001 | 0.45 | SPMSQ | Dairy products | Age and study levels | All | 0.801 |
| Dairy products | 0 | 0.001 | 0.429 | SPMSQ | Dairy products | Age, study levels and sex | All | 0.8 |
| Dairy products | 0.001 | 0.001 | 0.425 | SPMSQ | Dairy products | Age and study levels | Fem | 0.8 |
| Dairy products | 0 | 0.002 | 0.953 | SPMSQ | Dairy products | Age and study levels | Masc | 0.977 |
| Dairy products, masc sex | -0.001 | 0.002 | 0.784 | SPMSQ | Dairy products, sex | Age and study levels | All | 0.949 |
| Dairy products | -0.001 | 0.001 | 0.09 | MIS | Dairy products | Age and study levels | Fem | 0.603 |
| Dairy products | -0.001 | 0.001 | 0.105 | MIS | Dairy products | Age and study levels | All | 0.617 |
| Dairy products | -0.001 | 0.001 | 0.106 | MIS | Dairy products | Age, study levels and sex | All | 0.617 |
| Dairy products | -0.001 | 0.002 | 0.791 | MIS | Dairy products | Age and study levels | Masc | 0.949 |
| Dairy products, masc sex | 0.001 | 0.003 | 0.728 | MIS | Dairy products, sex | Age and study levels | All | 0.949 |
| Dairy products | -0.025 | 0.011 | 0.022 | SVF | Dairy products | Age and study levels | Masc | 0.536 |
| Dairy products | -0.005 | 0.003 | 0.095 | SVF | Dairy products | Age and study levels | All | 0.603 |
| Dairy products | -0.005 | 0.003 | 0.089 | SVF | Dairy products | Age, study levels and sex | All | 0.603 |
| Dairy products | -0.004 | 0.003 | 0.298 | SVF | Dairy products | Age and study levels | Fem | 0.74 |
| Dairy products, masc sex | -0.021 | 0.011 | 0.054 | SVF | Dairy products, sex | Age and study levels | All | 0.601 |
| Dark Chocolate | 0.049 | 0.023 | 0.041 | SPMSQ | Dark Chocolate | Age and study levels | Masc | 0.601 |
| Dark Chocolate | 0.016 | 0.014 | 0.244 | SPMSQ | Dark Chocolate | Age, study levels and sex | All | 0.721 |
| Dark Chocolate | 0.012 | 0.014 | 0.404 | SPMSQ | Dark Chocolate | Age and study levels | All | 0.784 |
| Dark Chocolate | 0.006 | 0.017 | 0.725 | SPMSQ | Dark Chocolate | Age and study levels | Fem | 0.949 |
| Dark Chocolate, masc sex | 0.043 | 0.032 | 0.186 | SPMSQ | Dark Chocolate, sex | Age and study levels | All | 0.691 |
| Dark Chocolate | -0.026 | 0.033 | 0.433 | MIS | Dark Chocolate | Age and study levels | Masc | 0.8 |
| Dark Chocolate | -0.005 | 0.017 | 0.77 | MIS | Dark Chocolate | Age and study levels | All | 0.949 |
| Dark Chocolate | -0.004 | 0.018 | 0.799 | MIS | Dark Chocolate | Age, study levels and sex | All | 0.949 |
| Dark Chocolate | 0.003 | 0.021 | 0.881 | MIS | Dark Chocolate | Age and study levels | Fem | 0.961 |
| Dark Chocolate, masc sex | -0.027 | 0.04 | 0.504 | MIS | Dark Chocolate, sex | Age and study levels | All | 0.826 |
| Dark Chocolate | 0.311 | 0.083 | 0 | SVF | Dark Chocolate | Age and study levels | Fem | 0 |
| Dark Chocolate | 0.248 | 0.072 | 0.001 | SVF | Dark Chocolate | Age and study levels | All | 0.065 |
| Dark Chocolate | 0.233 | 0.073 | 0.001 | SVF | Dark Chocolate | Age, study levels and sex | All | 0.065 |
| Dark Chocolate | 0.012 | 0.153 | 0.938 | SVF | Dark Chocolate | Age and study levels | Masc | 0.97 |
| Dark Chocolate, masc sex | -0.306 | 0.167 | 0.067 | SVF | Dark Chocolate, sex | Age and study levels | All | 0.603 |
| Enzymatic fruits | -0.007 | 0.002 | 0.005 | SPMSQ | Enzymatic fruits | Age and study levels | Masc | 0.244 |
| Enzymatic fruits | 0 | 0.001 | 0.759 | SPMSQ | Enzymatic fruits | Age, study levels and sex | All | 0.949 |
| Enzymatic fruits | 0 | 0.001 | 0.758 | SPMSQ | Enzymatic fruits | Age and study levels | Fem | 0.949 |
| Enzymatic fruits | 0 | 0.001 | 0.981 | SPMSQ | Enzymatic fruits | Age and study levels | All | 0.989 |
| Enzymatic fruits, masc sex | -0.007 | 0.003 | 0.018 | SPMSQ | Enzymatic fruits, sex | Age and study levels | All | 0.536 |
| Enzymatic fruits | 0.005 | 0.003 | 0.126 | MIS | Enzymatic fruits | Age and study levels | Masc | 0.637 |
| Enzymatic fruits | 0.001 | 0.001 | 0.139 | MIS | Enzymatic fruits | Age and study levels | All | 0.645 |
| Enzymatic fruits | 0.001 | 0.001 | 0.146 | MIS | Enzymatic fruits | Age, study levels and sex | All | 0.654 |
| Enzymatic fruits | 0.001 | 0.001 | 0.23 | MIS | Enzymatic fruits | Age and study levels | Fem | 0.721 |
| Enzymatic fruits, masc sex | 0.003 | 0.003 | 0.423 | MIS | Enzymatic fruits, sex | Age and study levels | All | 0.8 |
| Enzymatic fruits | 0.009 | 0.004 | 0.021 | SVF | Enzymatic fruits | Age and study levels | Fem | 0.536 |
| Enzymatic fruits | 0.007 | 0.004 | 0.056 | SVF | Enzymatic fruits | Age and study levels | All | 0.601 |
| Enzymatic fruits | 0.008 | 0.004 | 0.031 | SVF | Enzymatic fruits | Age, study levels and sex | All | 0.601 |
| Enzymatic fruits | -0.005 | 0.015 | 0.726 | SVF | Enzymatic fruits | Age and study levels | Masc | 0.949 |
| Enzymatic fruits, masc sex | -0.013 | 0.014 | 0.369 | SVF | Enzymatic fruits, sex | Age and study levels | All | 0.759 |
| Gluten free fods | -0.012 | 0.013 | 0.33 | SPMSQ | Gluten free fods | Age and study levels | Masc | 0.758 |
| Gluten free fods | 0.001 | 0.003 | 0.603 | SPMSQ | Gluten free fods | Age and study levels | All | 0.898 |
| Gluten free fods | 0.001 | 0.003 | 0.619 | SPMSQ | Gluten free fods | Age and study levels | Fem | 0.898 |
| Gluten free fods | 0.001 | 0.003 | 0.731 | SPMSQ | Gluten free fods | Age, study levels and sex | All | 0.949 |
| Gluten free fods, masc sex | -0.014 | 0.015 | 0.36 | SPMSQ | Gluten free fods, sex | Age and study levels | All | 0.759 |
| Gluten free fods | -0.003 | 0.003 | 0.364 | MIS | Gluten free fods | Age, study levels and sex | All | 0.759 |
| Gluten free fods | -0.003 | 0.003 | 0.377 | MIS | Gluten free fods | Age and study levels | All | 0.764 |
| Gluten free fods | -0.015 | 0.017 | 0.401 | MIS | Gluten free fods | Age and study levels | Masc | 0.784 |
| Gluten free fods | -0.003 | 0.003 | 0.456 | MIS | Gluten free fods | Age and study levels | Fem | 0.801 |
| Gluten free fods, masc sex | -0.013 | 0.019 | 0.501 | MIS | Gluten free fods, sex | Age and study levels | All | 0.824 |
| Gluten free fods | 0.012 | 0.014 | 0.373 | SVF | Gluten free fods | Age and study levels | Fem | 0.762 |
| Gluten free fods | 0.012 | 0.014 | 0.379 | SVF | Gluten free fods | Age, study levels and sex | All | 0.764 |
| Gluten free fods | 0.01 | 0.014 | 0.465 | SVF | Gluten free fods | Age and study levels | All | 0.808 |
| Gluten free fods | 0.012 | 0.08 | 0.884 | SVF | Gluten free fods | Age and study levels | Masc | 0.961 |
| Gluten free fods, masc sex | 0.001 | 0.078 | 0.993 | SVF | Gluten free fods, sex | Age and study levels | All | 0.996 |
| Non-greens | -0.004 | 0.003 | 0.13 | SPMSQ | Non-greens | Age and study levels | Masc | 0.637 |
| Non-greens | -0.001 | 0.001 | 0.444 | SPMSQ | Non-greens | Age, study levels and sex | All | 0.801 |
| Non-greens | -0.001 | 0.001 | 0.598 | SPMSQ | Non-greens | Age and study levels | All | 0.898 |
| Non-greens | 0 | 0.001 | 0.753 | SPMSQ | Non-greens | Age and study levels | Fem | 0.949 |
| Non-greens, masc sex | -0.004 | 0.003 | 0.27 | SPMSQ | Non-greens, sex | Age and study levels | All | 0.734 |
| Non-greens | 0.009 | 0.003 | 0.013 | MIS | Non-greens | Age and study levels | Masc | 0.536 |
| Non-greens | -0.001 | 0.001 | 0.347 | MIS | Non-greens | Age and study levels | Fem | 0.759 |
| Non-greens | 0 | 0.001 | 0.889 | MIS | Non-greens | Age, study levels and sex | All | 0.961 |
| Non-greens | 0 | 0.001 | 0.913 | MIS | Non-greens | Age and study levels | All | 0.968 |
| Non-greens, masc sex | 0.009 | 0.004 | 0.02 | MIS | Non-greens, sex | Age and study levels | All | 0.536 |
| Non-greens | -0.009 | 0.005 | 0.092 | SVF | Non-greens | Age and study levels | All | 0.603 |
| Non-greens | -0.01 | 0.006 | 0.099 | SVF | Non-greens | Age and study levels | Fem | 0.603 |
| Non-greens | -0.008 | 0.005 | 0.131 | SVF | Non-greens | Age, study levels and sex | All | 0.637 |
| Non-greens | 0.001 | 0.017 | 0.961 | SVF | Non-greens | Age and study levels | Masc | 0.977 |
| Non-greens, masc sex | 0.01 | 0.017 | 0.565 | SVF | Non-greens, sex | Age and study levels | All | 0.881 |
| Other fruits | -0.002 | 0.002 | 0.31 | SPMSQ | Other fruits | Age and study levels | Masc | 0.751 |
| Other fruits | 0 | 0.001 | 0.65 | SPMSQ | Other fruits | Age and study levels | All | 0.911 |
| Other fruits | 0 | 0.001 | 0.825 | SPMSQ | Other fruits | Age, study levels and sex | All | 0.949 |
| Other fruits | 0 | 0.001 | 0.89 | SPMSQ | Other fruits | Age and study levels | Fem | 0.961 |
| Other fruits, masc sex | -0.003 | 0.003 | 0.369 | SPMSQ | Other fruits, sex | Age and study levels | All | 0.759 |
| Other fruits | 0.002 | 0.001 | 0.089 | MIS | Other fruits | Age and study levels | All | 0.603 |
| Other fruits | 0.002 | 0.001 | 0.082 | MIS | Other fruits | Age, study levels and sex | All | 0.603 |
| Other fruits | 0.002 | 0.001 | 0.134 | MIS | Other fruits | Age and study levels | Fem | 0.637 |
| Other fruits | 0.004 | 0.003 | 0.241 | MIS | Other fruits | Age and study levels | Masc | 0.721 |
| Other fruits, masc sex | 0.001 | 0.004 | 0.776 | MIS | Other fruits, sex | Age and study levels | All | 0.949 |
| Other fruits | -0.011 | 0.015 | 0.462 | SVF | Other fruits | Age and study levels | Masc | 0.808 |
| Other fruits | -0.002 | 0.005 | 0.666 | SVF | Other fruits | Age, study levels and sex | All | 0.921 |
| Other fruits | -0.001 | 0.005 | 0.809 | SVF | Other fruits | Age and study levels | All | 0.949 |
| Other fruits | -0.001 | 0.006 | 0.851 | SVF | Other fruits | Age and study levels | Fem | 0.959 |
| Other fruits, masc sex | -0.009 | 0.015 | 0.53 | SVF | Other fruits, sex | Age and study levels | All | 0.856 |
| Other Vegetables | -0.002 | 0.002 | 0.481 | SPMSQ | Other Vegetables | Age and study levels | Masc | 0.812 |
| Other Vegetables | -0.001 | 0.001 | 0.551 | SPMSQ | Other Vegetables | Age, study levels and sex | All | 0.87 |
| Other Vegetables | -0.001 | 0.001 | 0.582 | SPMSQ | Other Vegetables | Age and study levels | All | 0.894 |
| Other Vegetables | 0 | 0.001 | 0.708 | SPMSQ | Other Vegetables | Age and study levels | Fem | 0.949 |
| Other Vegetables, masc sex | -0.001 | 0.003 | 0.613 | SPMSQ | Other Vegetables, sex | Age and study levels | All | 0.898 |
| Other Vegetables | 0.004 | 0.003 | 0.243 | MIS | Other Vegetables | Age and study levels | Masc | 0.721 |
| Other Vegetables | 0.001 | 0.001 | 0.593 | MIS | Other Vegetables | Age and study levels | All | 0.898 |
| Other Vegetables | 0.001 | 0.001 | 0.597 | MIS | Other Vegetables | Age, study levels and sex | All | 0.898 |
| Other Vegetables | 0 | 0.001 | 0.807 | MIS | Other Vegetables | Age and study levels | Fem | 0.949 |
| Other Vegetables, masc sex | 0.003 | 0.004 | 0.485 | MIS | Other Vegetables, sex | Age and study levels | All | 0.812 |
| Other Vegetables | -0.006 | 0.015 | 0.681 | SVF | Other Vegetables | Age and study levels | Masc | 0.937 |
| Other Vegetables | -0.001 | 0.005 | 0.906 | SVF | Other Vegetables | Age and study levels | All | 0.963 |
| Other Vegetables | 0 | 0.005 | 0.932 | SVF | Other Vegetables | Age, study levels and sex | All | 0.97 |
| Other Vegetables | 0 | 0.005 | 0.966 | SVF | Other Vegetables | Age and study levels | Fem | 0.977 |
| Other Vegetables, masc sex | -0.006 | 0.015 | 0.715 | SVF | Other Vegetables, sex | Age and study levels | All | 0.949 |
| Plain yogurt or kefir | -0.001 | 0.001 | 0.286 | SPMSQ | Plain yogurt or kefir | Age, study levels and sex | All | 0.734 |
| Plain yogurt or kefir | -0.001 | 0.001 | 0.332 | SPMSQ | Plain yogurt or kefir | Age and study levels | Fem | 0.758 |
| Plain yogurt or kefir | 0.003 | 0.003 | 0.335 | SVF | Plain yogurt or kefir | Age, study levels and sex | All | 0.758 |
| Plain yogurt or kefir | 0.003 | 0.003 | 0.326 | SVF | Plain yogurt or kefir | Age and study levels | Fem | 0.758 |
| Plain yogurt or kefir | -0.001 | 0.001 | 0.37 | SPMSQ | Plain yogurt or kefir | Age and study levels | All | 0.759 |
| Plain yogurt or kefir | 0.003 | 0.003 | 0.406 | SVF | Plain yogurt or kefir | Age and study levels | All | 0.784 |
| Plain yogurt or kefir | -0.001 | 0.003 | 0.622 | SPMSQ | Plain yogurt or kefir | Age and study levels | Masc | 0.898 |
| Plain yogurt or kefir | -0.001 | 0.004 | 0.803 | MIS | Plain yogurt or kefir | Age and study levels | Masc | 0.949 |
| Plain yogurt or kefir | 0 | 0.001 | 0.844 | MIS | Plain yogurt or kefir | Age and study levels | Fem | 0.959 |
| Plain yogurt or kefir | 0 | 0.001 | 0.884 | MIS | Plain yogurt or kefir | Age and study levels | All | 0.961 |
| Plain yogurt or kefir | 0 | 0.001 | 0.899 | MIS | Plain yogurt or kefir | Age, study levels and sex | All | 0.962 |
| Plain yogurt or kefir | 0 | 0.017 | 0.991 | SVF | Plain yogurt or kefir | Age and study levels | Masc | 0.996 |
| Plain yogurt or kefir, masc sex | -0.001 | 0.004 | 0.732 | MIS | Plain yogurt or kefir, sex | Age and study levels | All | 0.949 |
| Plain yogurt or kefir, masc sex | -0.001 | 0.003 | 0.821 | SPMSQ | Plain yogurt or kefir, sex | Age and study levels | All | 0.949 |
| Plain yogurt or kefir, masc sex | -0.003 | 0.016 | 0.837 | SVF | Plain yogurt or kefir, sex | Age and study levels | All | 0.956 |
| Red Meat | -0.036 | 0.018 | 0.044 | SVF | Red Meat | Age and study levels | All | 0.601 |
| Red Meat | -0.036 | 0.018 | 0.045 | SVF | Red Meat | Age, study levels and sex | All | 0.601 |
| Red Meat | -0.037 | 0.018 | 0.043 | SVF | Red Meat | Age and study levels | Fem | 0.601 |
| Red Meat | 0.006 | 0.003 | 0.091 | SPMSQ | Red Meat | Age and study levels | All | 0.603 |
| Red Meat | 0.006 | 0.003 | 0.093 | SPMSQ | Red Meat | Age, study levels and sex | All | 0.603 |
| Red Meat | 0.006 | 0.004 | 0.113 | SPMSQ | Red Meat | Age and study levels | Fem | 0.626 |
| Red Meat | 0.01 | 0.015 | 0.494 | MIS | Red Meat | Age and study levels | Masc | 0.816 |
| Red Meat | -0.002 | 0.004 | 0.592 | MIS | Red Meat | Age and study levels | Fem | 0.898 |
| Red Meat | 0.006 | 0.011 | 0.613 | SPMSQ | Red Meat | Age and study levels | Masc | 0.898 |
| Red Meat | -0.001 | 0.004 | 0.737 | MIS | Red Meat | Age and study levels | All | 0.949 |
| Red Meat | -0.001 | 0.004 | 0.735 | MIS | Red Meat | Age, study levels and sex | All | 0.949 |
| Red Meat | -0.013 | 0.07 | 0.847 | SVF | Red Meat | Age and study levels | Masc | 0.959 |
| Red Meat, masc sex | 0.013 | 0.017 | 0.432 | MIS | Red Meat, sex | Age and study levels | All | 0.8 |
| Red Meat, masc sex | 0.021 | 0.069 | 0.764 | SVF | Red Meat, sex | Age and study levels | All | 0.949 |
| Red Meat, masc sex | 0 | 0.013 | 0.996 | SPMSQ | Red Meat, sex | Age and study levels | All | 0.996 |
| Refined Grains | 0.022 | 0.007 | 0.003 | MIS | Refined Grains | Age and study levels | Masc | 0.167 |
| Refined Grains | -0.022 | 0.009 | 0.015 | SVF | Refined Grains | Age and study levels | All | 0.536 |
| Refined Grains | -0.019 | 0.009 | 0.04 | SVF | Refined Grains | Age, study levels and sex | All | 0.601 |
| Refined Grains | -0.02 | 0.01 | 0.038 | SVF | Refined Grains | Age and study levels | Fem | 0.601 |
| Refined Grains | 0.004 | 0.002 | 0.086 | MIS | Refined Grains | Age and study levels | All | 0.603 |
| Refined Grains | 0.004 | 0.002 | 0.092 | MIS | Refined Grains | Age, study levels and sex | All | 0.603 |
| Refined Grains | -0.009 | 0.005 | 0.123 | SPMSQ | Refined Grains | Age and study levels | Masc | 0.636 |
| Refined Grains | 0.002 | 0.002 | 0.362 | MIS | Refined Grains | Age and study levels | Fem | 0.759 |
| Refined Grains | -0.002 | 0.002 | 0.365 | SPMSQ | Refined Grains | Age, study levels and sex | All | 0.759 |
| Refined Grains | -0.001 | 0.002 | 0.559 | SPMSQ | Refined Grains | Age and study levels | Fem | 0.876 |
| Refined Grains | -0.001 | 0.002 | 0.73 | SPMSQ | Refined Grains | Age and study levels | All | 0.949 |
| Refined Grains | -0.012 | 0.036 | 0.745 | SVF | Refined Grains | Age and study levels | Masc | 0.949 |
| Refined Grains, masc sex | 0.02 | 0.008 | 0.016 | MIS | Refined Grains, sex | Age and study levels | All | 0.536 |
| Refined Grains, masc sex | -0.007 | 0.007 | 0.287 | SPMSQ | Refined Grains, sex | Age and study levels | All | 0.734 |
| Refined Grains, masc sex | 0.01 | 0.035 | 0.786 | SVF | Refined Grains, sex | Age and study levels | All | 0.949 |
| Sugary products | 0.01 | 0.005 | 0.037 | SPMSQ | Sugary products | Age and study levels | Fem | 0.601 |
| Sugary products | -0.043 | 0.022 | 0.047 | SVF | Sugary products | Age and study levels | All | 0.601 |
| Sugary products | 0.008 | 0.004 | 0.064 | SPMSQ | Sugary products | Age and study levels | All | 0.603 |
| Sugary products | -0.039 | 0.022 | 0.068 | SVF | Sugary products | Age, study levels and sex | All | 0.603 |
| Sugary products | 0.007 | 0.004 | 0.098 | SPMSQ | Sugary products | Age, study levels and sex | All | 0.603 |
| Sugary products | -0.037 | 0.023 | 0.111 | SVF | Sugary products | Age and study levels | Fem | 0.626 |
| Sugary products | 0.015 | 0.013 | 0.221 | MIS | Sugary products | Age and study levels | Masc | 0.721 |
| Sugary products | -0.009 | 0.009 | 0.333 | SPMSQ | Sugary products | Age and study levels | Masc | 0.758 |
| Sugary products | -0.054 | 0.058 | 0.352 | SVF | Sugary products | Age and study levels | Masc | 0.759 |
| Sugary products | -0.004 | 0.006 | 0.476 | MIS | Sugary products | Age and study levels | Fem | 0.811 |
| Sugary products | -0.001 | 0.005 | 0.884 | MIS | Sugary products | Age and study levels | All | 0.961 |
| Sugary products | -0.001 | 0.005 | 0.864 | MIS | Sugary products | Age, study levels and sex | All | 0.961 |
| Sugary products, masc sex | -0.019 | 0.012 | 0.109 | SPMSQ | Sugary products , sex | Age and study levels | All | 0.625 |
| Sugary products, masc sex | 0.019 | 0.014 | 0.185 | MIS | Sugary products, sex | Age and study levels | All | 0.691 |
| Sugary products, masc sex | -0.016 | 0.06 | 0.792 | SVF | Sugary products, sex | Age and study levels | All | 0.949 |
| Sweetened Beverages | -0.001 | 0.001 | 0.254 | MIS | Sweetened Beverages | Age and study levels | All | 0.721 |
| Sweetened Beverages | -0.001 | 0.001 | 0.245 | MIS | Sweetened Beverages | Age, study levels and sex | All | 0.721 |
| Sweetened Beverages | 0.001 | 0.001 | 0.221 | SPMSQ | Sweetened Beverages | Age and study levels | All | 0.721 |
| Sweetened Beverages | -0.001 | 0.001 | 0.286 | MIS | Sweetened Beverages | Age and study levels | Fem | 0.734 |
| Sweetened Beverages | 0.001 | 0.001 | 0.288 | SPMSQ | Sweetened Beverages | Age, study levels and sex | All | 0.734 |
| Sweetened Beverages | 0.001 | 0.001 | 0.292 | SPMSQ | Sweetened Beverages | Age and study levels | Fem | 0.735 |
| Sweetened Beverages | -0.006 | 0.006 | 0.326 | MIS | Sweetened Beverages | Age and study levels | Masc | 0.758 |
| Sweetened Beverages | -0.026 | 0.03 | 0.387 | SVF | Sweetened Beverages | Age and study levels | Masc | 0.774 |
| Sweetened Beverages | -0.003 | 0.005 | 0.492 | SVF | Sweetened Beverages | Age and study levels | All | 0.816 |
| Sweetened Beverages | -0.003 | 0.005 | 0.578 | SVF | Sweetened Beverages | Age, study levels and sex | All | 0.893 |
| Sweetened Beverages | -0.002 | 0.005 | 0.692 | SVF | Sweetened Beverages | Age and study levels | Fem | 0.947 |
| Sweetened Beverages | -0.001 | 0.005 | 0.871 | SPMSQ | Sweetened Beverages | Age and study levels | Masc | 0.961 |
| Sweetened Beverages, masc sex | -0.019 | 0.026 | 0.456 | SVF | Sweetened Beverages, sex | Age and study levels | All | 0.801 |
| Sweetened Beverages, masc sex | -0.002 | 0.006 | 0.8 | MIS | Sweetened Beverages, sex | Age and study levels | All | 0.949 |
| Sweetened Beverages, masc sex | -0.001 | 0.005 | 0.892 | SPMSQ | Sweetened Beverages, sex | Age and study levels | All | 0.961 |
| Plant-based milk substitutes | 0.033 | 0.022 | 0.139 | SVF | Vegetable Drinks | Age and study levels | Masc | 0.645 |
| Plant-based milk substitutes | 0.005 | 0.004 | 0.177 | SVF | Vegetable Drinks | Age, study levels and sex | All | 0.677 |
| Plant-based milk substitutes | 0.004 | 0.004 | 0.272 | SVF | Vegetable Drinks | Age and study levels | All | 0.734 |
| Plant-based milk substitutes | 0.004 | 0.004 | 0.264 | SVF | Vegetable Drinks | Age and study levels | Fem | 0.734 |
| Plant-based milk substitutes | -0.004 | 0.005 | 0.433 | MIS | Vegetable Drinks | Age and study levels | Masc | 0.8 |
| Plant-based milk substitutes | -0.003 | 0.003 | 0.466 | SPMSQ | Vegetable Drinks | Age and study levels | Masc | 0.808 |
| Plant-based milk substitutes | 0 | 0.001 | 0.645 | MIS | Vegetable Drinks | Age and study levels | Fem | 0.911 |
| Plant-based milk substitutes | 0 | 0.001 | 0.719 | SPMSQ | Vegetable Drinks | Age, study levels and sex | All | 0.949 |
| Plant-based milk substitutes | 0 | 0.001 | 0.757 | MIS | Vegetable Drinks | Age and study levels | All | 0.949 |
| Plant-based milk substitutes | 0 | 0.001 | 0.785 | MIS | Vegetable Drinks | Age, study levels and sex | All | 0.949 |
| Plant-based milk substitutes | 0 | 0.001 | 0.818 | SPMSQ | Vegetable Drinks | Age and study levels | Fem | 0.949 |
| Plant-based milk substitutes | 0 | 0.001 | 0.955 | SPMSQ | Vegetable Drinks | Age and study levels | All | 0.977 |
| Plant-based milk substitutes, masc sex | 0.026 | 0.021 | 0.215 | SVF | Vegetable Drinks, sex | Age and study levels | All | 0.721 |
| Plant-based milk substitutes, masc sex | -0.004 | 0.005 | 0.446 | MIS | Vegetable Drinks, sex | Age and study levels | All | 0.801 |
| Plant-based milk substitutes, masc sex | -0.002 | 0.004 | 0.593 | SPMSQ | Vegetable Drinks, sex | Age and study levels | All | 0.898 |
| White Fish | 0.019 | 0.012 | 0.118 | SPMSQ | White Fish | Age and study levels | Masc | 0.63 |
| White Fish | -0.031 | 0.024 | 0.196 | SVF | White Fish | Age and study levels | All | 0.701 |
| White Fish | -0.029 | 0.024 | 0.228 | SVF | White Fish | Age, study levels and sex | All | 0.721 |
| White Fish | -0.079 | 0.078 | 0.31 | SVF | White Fish | Age and study levels | Masc | 0.751 |
| White Fish | -0.024 | 0.025 | 0.355 | SVF | White Fish | Age and study levels | Fem | 0.759 |
| White Fish | -0.003 | 0.005 | 0.514 | SPMSQ | White Fish | Age and study levels | Fem | 0.839 |
| White Fish | 0.004 | 0.006 | 0.57 | MIS | White Fish | Age and study levels | Fem | 0.886 |
| White Fish | -0.01 | 0.017 | 0.579 | MIS | White Fish | Age and study levels | Masc | 0.893 |
| White Fish | 0.002 | 0.006 | 0.71 | MIS | White Fish | Age and study levels | All | 0.949 |
| White Fish | 0.002 | 0.006 | 0.72 | MIS | White Fish | Age, study levels and sex | All | 0.949 |
| White Fish | -0.001 | 0.005 | 0.832 | SPMSQ | White Fish | Age, study levels and sex | All | 0.954 |
| White Fish | 0 | 0.005 | 0.925 | SPMSQ | White Fish | Age and study levels | All | 0.97 |
| White Fish, masc sex | 0.02 | 0.015 | 0.176 | SPMSQ | White Fish, sex | Age and study levels | All | 0.677 |
| White Fish, masc sex | -0.015 | 0.018 | 0.406 | MIS | White Fish, sex | Age and study levels | All | 0.784 |
| White Fish, masc sex | -0.055 | 0.077 | 0.475 | SVF | White Fish, sex | Age and study levels | All | 0.811 |
| White Meat | 0.01 | 0.009 | 0.275 | MIS | White Meat | Age and study levels | Masc | 0.734 |
| White Meat | -0.003 | 0.003 | 0.39 | SPMSQ | White Meat | Age and study levels | Fem | 0.776 |
| White Meat | -0.002 | 0.003 | 0.453 | SPMSQ | White Meat | Age and study levels | All | 0.801 |
| White Meat | -0.002 | 0.003 | 0.437 | SPMSQ | White Meat | Age, study levels and sex | All | 0.801 |
| White Meat | -0.011 | 0.015 | 0.475 | SVF | White Meat | Age and study levels | All | 0.811 |
| White Meat | -0.011 | 0.015 | 0.485 | SVF | White Meat | Age, study levels and sex | All | 0.812 |
| White Meat | -0.01 | 0.016 | 0.538 | SVF | White Meat | Age and study levels | Fem | 0.863 |
| White Meat | 0.002 | 0.004 | 0.605 | MIS | White Meat | Age and study levels | All | 0.898 |
| White Meat | 0.002 | 0.004 | 0.607 | MIS | White Meat | Age, study levels and sex | All | 0.898 |
| White Meat | -0.019 | 0.042 | 0.653 | SVF | White Meat | Age and study levels | Masc | 0.911 |
| White Meat | 0 | 0.004 | 0.905 | MIS | White Meat | Age and study levels | Fem | 0.963 |
| White Meat | 0.001 | 0.007 | 0.92 | SPMSQ | White Meat | Age and study levels | Masc | 0.968 |
| White Meat, masc sex | 0.01 | 0.01 | 0.327 | MIS | White Meat, sex | Age and study levels | All | 0.758 |
| White Meat, masc sex | 0.004 | 0.008 | 0.654 | SPMSQ | White Meat, sex | Age and study levels | All | 0.911 |
| White Meat, masc sex | -0.006 | 0.043 | 0.895 | SVF | White Meat, sex | Age and study levels | All | 0.962 |
| Berries | -0.002 | 0.001 | 0.053 | SPMSQ | Berries | Age and study levels | All | 0.601 |
| Berries | -0.001 | 0.001 | 0.089 | SPMSQ | Berries | Age, study levels and sex | All | 0.603 |
| Berries | 0.009 | 0.005 | 0.075 | SVF | Berries | Age and study levels | Fem | 0.603 |
| Berries | -0.002 | 0.001 | 0.114 | SPMSQ | Berries | Age and study levels | Masc | 0.626 |
| Berries | -0.002 | 0.001 | 0.132 | MIS | Berries | Age and study levels | Fem | 0.637 |
| Berries | 0.006 | 0.004 | 0.133 | SVF | Berries | Age and study levels | All | 0.637 |
| Berries | 0.006 | 0.004 | 0.188 | SVF | Berries | Age, study levels and sex | All | 0.692 |
| Berries | -0.001 | 0.001 | 0.244 | MIS | Berries | Age and study levels | All | 0.721 |
| Berries | -0.001 | 0.001 | 0.255 | MIS | Berries | Age, study levels and sex | All | 0.721 |
| Berries | -0.001 | 0.001 | 0.254 | SPMSQ | Berries | Age and study levels | Fem | 0.721 |
| Berries | 0.001 | 0.002 | 0.742 | MIS | Berries | Age and study levels | Masc | 0.949 |
| Berries | -0.002 | 0.008 | 0.85 | SVF | Berries | Age and study levels | Masc | 0.959 |
| Berries, masc sex | -0.011 | 0.01 | 0.243 | SVF | Berries, sex | Age and study levels | All | 0.721 |
| Berries, masc sex | 0.002 | 0.002 | 0.276 | MIS | Berries, sex | Age and study levels | All | 0.734 |
| Berries, masc sex | -0.001 | 0.002 | 0.638 | SPMSQ | Berries, sex | Age and study levels | All | 0.908 |
| Coffee | -0.014 | 0.008 | 0.097 | MIS | Coffee | Age and study levels | Fem | 0.603 |
| Coffee | 0.031 | 0.02 | 0.117 | SPMSQ | Coffee | Age and study levels | Masc | 0.63 |
| Coffee | -0.186 | 0.126 | 0.144 | SVF | Coffee | Age and study levels | Masc | 0.654 |
| Coffee | -0.011 | 0.008 | 0.154 | MIS | Coffee | Age and study levels | All | 0.658 |
| Coffee | -0.011 | 0.008 | 0.157 | MIS | Coffee | Age, study levels and sex | All | 0.658 |
| Coffee | 0.047 | 0.034 | 0.167 | SVF | Coffee | Age and study levels | Fem | 0.677 |
| Coffee | 0.031 | 0.033 | 0.345 | SVF | Coffee | Age and study levels | All | 0.759 |
| Coffee | 0.029 | 0.033 | 0.369 | SVF | Coffee | Age, study levels and sex | All | 0.759 |
| Coffee | 0.019 | 0.028 | 0.488 | MIS | Coffee | Age and study levels | Masc | 0.813 |
| Coffee | 0.001 | 0.006 | 0.824 | SPMSQ | Coffee | Age, study levels and sex | All | 0.949 |
| Coffee | 0.001 | 0.006 | 0.872 | SPMSQ | Coffee | Age and study levels | All | 0.961 |
| Coffee | -0.001 | 0.007 | 0.886 | SPMSQ | Coffee | Age and study levels | Fem | 0.961 |
| Coffee, masc sex | -0.23 | 0.126 | 0.067 | SVF | Coffee, sex | Age and study levels | All | 0.603 |
| Coffee, masc sex | 0.033 | 0.024 | 0.177 | SPMSQ | Coffee, sex | Age and study levels | All | 0.677 |
| Coffee, masc sex | 0.036 | 0.03 | 0.234 | MIS | Coffee, sex | Age and study levels | All | 0.721 |
| Eggs | 0.072 | 0.019 | 0 | SVF | Eggs | Age and study levels | All | 0 |
| Eggs | 0.069 | 0.019 | 0 | SVF | Eggs | Age, study levels and sex | All | 0 |
| Eggs | 0.07 | 0.02 | 0 | SVF | Eggs | Age and study levels | Fem | 0 |
| Eggs | 0.027 | 0.013 | 0.049 | MIS | Eggs | Age and study levels | Masc | 0.601 |
| Eggs | -0.005 | 0.004 | 0.194 | SPMSQ | Eggs | Age and study levels | All | 0.701 |
| Eggs | -0.004 | 0.004 | 0.287 | SPMSQ | Eggs | Age, study levels and sex | All | 0.734 |
| Eggs | -0.004 | 0.004 | 0.351 | SPMSQ | Eggs | Age and study levels | Fem | 0.759 |
| Eggs | 0.053 | 0.063 | 0.399 | SVF | Eggs | Age and study levels | Masc | 0.784 |
| Eggs | -0.006 | 0.01 | 0.543 | SPMSQ | Eggs | Age and study levels | Masc | 0.868 |
| Eggs | 0.002 | 0.005 | 0.626 | MIS | Eggs | Age, study levels and sex | All | 0.901 |
| Eggs | 0.002 | 0.005 | 0.649 | MIS | Eggs | Age and study levels | All | 0.911 |
| Eggs | -0.001 | 0.005 | 0.917 | MIS | Eggs | Age and study levels | Fem | 0.968 |
| Eggs, masc sex | 0.025 | 0.015 | 0.093 | MIS | Eggs, sex | Age and study levels | All | 0.603 |
| Eggs, masc sex | -0.003 | 0.012 | 0.816 | SPMSQ | Eggs, sex | Age and study levels | All | 0.949 |
| Eggs, masc sex | -0.017 | 0.062 | 0.786 | SVF | Eggs, sex | Age and study levels | All | 0.949 |
| EVOO | 0.014 | 0.006 | 0.022 | SVF | EVOO | Age and study levels | All | 0.536 |
| EVOO | 0.013 | 0.006 | 0.036 | SVF | EVOO | Age, study levels and sex | All | 0.601 |
| EVOO | 0.013 | 0.006 | 0.038 | SVF | EVOO | Age and study levels | Fem | 0.601 |
| EVOO | -0.006 | 0.004 | 0.124 | MIS | EVOO | Age and study levels | Masc | 0.636 |
| EVOO | -0.002 | 0.001 | 0.156 | SPMSQ | EVOO | Age and study levels | Fem | 0.658 |
| EVOO | -0.002 | 0.001 | 0.191 | SPMSQ | EVOO | Age and study levels | All | 0.696 |
| EVOO | 0.002 | 0.002 | 0.277 | MIS | EVOO | Age and study levels | Fem | 0.734 |
| EVOO | -0.001 | 0.001 | 0.288 | SPMSQ | EVOO | Age, study levels and sex | All | 0.734 |
| EVOO | 0.003 | 0.003 | 0.338 | SPMSQ | EVOO | Age and study levels | Masc | 0.758 |
| EVOO | 0.016 | 0.019 | 0.38 | SVF | EVOO | Age and study levels | Masc | 0.764 |
| EVOO | 0.001 | 0.001 | 0.63 | MIS | EVOO | Age, study levels and sex | All | 0.903 |
| EVOO | 0.001 | 0.001 | 0.654 | MIS | EVOO | Age and study levels | All | 0.911 |
| EVOO, masc sex | -0.007 | 0.004 | 0.083 | MIS | EVOO, sex | Age and study levels | All | 0.603 |
| EVOO, masc sex | 0.004 | 0.003 | 0.219 | SPMSQ | EVOO, sex | Age and study levels | All | 0.721 |
| EVOO, masc sex | -0.004 | 0.017 | 0.824 | SVF | EVOO, sex | Age and study levels | All | 0.949 |
| Greens | -0.003 | 0.002 | 0.18 | SPMSQ | Greens | Age and study levels | Masc | 0.682 |
| Greens | -0.001 | 0.001 | 0.199 | SPMSQ | Greens | Age, study levels and sex | All | 0.706 |
| Greens | -0.001 | 0.001 | 0.339 | SPMSQ | Greens | Age and study levels | Fem | 0.758 |
| Greens | -0.001 | 0.001 | 0.355 | SPMSQ | Greens | Age and study levels | All | 0.759 |
| Greens | -0.002 | 0.004 | 0.657 | SVF | Greens | Age and study levels | All | 0.912 |
| Greens | 0.001 | 0.003 | 0.782 | MIS | Greens | Age and study levels | Masc | 0.949 |
| Greens | -0.001 | 0.004 | 0.773 | SVF | Greens | Age and study levels | Fem | 0.949 |
| Greens | 0.003 | 0.012 | 0.791 | SVF | Greens | Age and study levels | Masc | 0.949 |
| Greens | -0.001 | 0.004 | 0.863 | SVF | Greens | Age, study levels and sex | All | 0.961 |
| Greens | 0 | 0.001 | 0.921 | MIS | Greens | Age and study levels | All | 0.968 |
| Greens | 0 | 0.001 | 0.945 | MIS | Greens | Age and study levels | Fem | 0.975 |
| Greens | 0 | 0.001 | 0.955 | MIS | Greens | Age, study levels and sex | All | 0.977 |
| Greens, masc sex | -0.002 | 0.002 | 0.453 | SPMSQ | Greens, sex | Age and study levels | All | 0.801 |
| Greens, masc sex | 0.001 | 0.003 | 0.825 | MIS | Greens, sex | Age and study levels | All | 0.949 |
| Greens, masc sex | 0.003 | 0.012 | 0.781 | SVF | Greens, sex | Age and study levels | All | 0.949 |
| Legumes | -0.042 | 0.024 | 0.082 | SVF | Legumes | Age and study levels | Fem | 0.603 |
| Legumes | -0.032 | 0.022 | 0.146 | SVF | Legumes | Age, study levels and sex | All | 0.654 |
| Legumes | 0.008 | 0.006 | 0.148 | MIS | Legumes | Age and study levels | Fem | 0.656 |
| Legumes | -0.031 | 0.022 | 0.162 | SVF | Legumes | Age and study levels | All | 0.669 |
| Legumes | 0.006 | 0.005 | 0.251 | MIS | Legumes | Age and study levels | All | 0.721 |
| Legumes | 0.006 | 0.005 | 0.248 | MIS | Legumes | Age, study levels and sex | All | 0.721 |
| Legumes | -0.006 | 0.01 | 0.519 | SPMSQ | Legumes | Age and study levels | Masc | 0.843 |
| Legumes | 0.002 | 0.005 | 0.612 | SPMSQ | Legumes | Age and study levels | Fem | 0.898 |
| Legumes | -0.005 | 0.013 | 0.734 | MIS | Legumes | Age and study levels | Masc | 0.949 |
| Legumes | 0.001 | 0.004 | 0.813 | SPMSQ | Legumes | Age, study levels and sex | All | 0.949 |
| Legumes | 0.017 | 0.061 | 0.786 | SVF | Legumes | Age and study levels | Masc | 0.949 |
| Legumes | 0.001 | 0.004 | 0.863 | SPMSQ | Legumes | Age and study levels | All | 0.961 |
| Legumes, masc sex | -0.014 | 0.015 | 0.34 | MIS | Legumes, sex | Age and study levels | All | 0.758 |
| Legumes, masc sex | 0.061 | 0.063 | 0.333 | SVF | Legumes, sex | Age and study levels | All | 0.758 |
| Legumes, masc sex | -0.009 | 0.012 | 0.455 | SPMSQ | Legumes, sex | Age and study levels | All | 0.801 |
| Nuts | 0.041 | 0.02 | 0.04 | SVF | Nuts | Age and study levels | All | 0.601 |
| Nuts | 0.038 | 0.02 | 0.056 | SVF | Nuts | Age, study levels and sex | All | 0.601 |
| Nuts | 0.04 | 0.021 | 0.059 | SVF | Nuts | Age and study levels | Fem | 0.603 |
| Nuts | -0.007 | 0.005 | 0.157 | MIS | Nuts | Age and study levels | All | 0.658 |
| Nuts | -0.007 | 0.005 | 0.163 | MIS | Nuts | Age, study levels and sex | All | 0.669 |
| Nuts | -0.017 | 0.015 | 0.247 | MIS | Nuts | Age and study levels | Masc | 0.721 |
| Nuts | -0.005 | 0.004 | 0.223 | SPMSQ | Nuts | Age and study levels | All | 0.721 |
| Nuts | -0.005 | 0.005 | 0.284 | MIS | Nuts | Age and study levels | Fem | 0.734 |
| Nuts | -0.004 | 0.004 | 0.302 | SPMSQ | Nuts | Age, study levels and sex | All | 0.745 |
| Nuts | -0.004 | 0.004 | 0.319 | SPMSQ | Nuts | Age and study levels | Fem | 0.758 |
| Nuts | 0.043 | 0.068 | 0.531 | SVF | Nuts | Age and study levels | Masc | 0.856 |
| Nuts | -0.003 | 0.011 | 0.805 | SPMSQ | Nuts | Age and study levels | Masc | 0.949 |
| Nuts, masc sex | -0.01 | 0.016 | 0.549 | MIS | Nuts, sex | Age and study levels | All | 0.87 |
| Nuts, masc sex | 0.002 | 0.013 | 0.857 | SPMSQ | Nuts, sex | Age and study levels | All | 0.961 |
| Nuts, masc sex | -0.009 | 0.066 | 0.888 | SVF | Nuts, sex | Age and study levels | All | 0.961 |
| Sauces | -0.089 | 0.078 | 0.254 | SVF | Sauces | Age and study levels | Fem | 0.721 |
| Sauces | -0.083 | 0.075 | 0.268 | SVF | Sauces | Age and study levels | All | 0.734 |
| Sauces | -0.081 | 0.075 | 0.282 | SVF | Sauces | Age, study levels and sex | All | 0.734 |
| Sauces | 0.07 | 0.066 | 0.291 | MIS | Sauces | Age and study levels | Masc | 0.735 |
| Sauces | 0.011 | 0.015 | 0.448 | SPMSQ | Sauces | Age and study levels | All | 0.801 |
| Sauces | 0.01 | 0.014 | 0.472 | SPMSQ | Sauces | Age, study levels and sex | All | 0.811 |
| Sauces | 0.029 | 0.048 | 0.546 | SPMSQ | Sauces | Age and study levels | Masc | 0.869 |
| Sauces | 0.009 | 0.015 | 0.554 | SPMSQ | Sauces | Age and study levels | Fem | 0.871 |
| Sauces | 0.006 | 0.018 | 0.719 | MIS | Sauces | Age and study levels | All | 0.949 |
| Sauces | 0.006 | 0.018 | 0.723 | MIS | Sauces | Age, study levels and sex | All | 0.949 |
| Sauces | 0.002 | 0.019 | 0.936 | MIS | Sauces | Age and study levels | Fem | 0.97 |
| Sauces | -0.014 | 0.305 | 0.964 | SVF | Sauces | Age and study levels | Masc | 0.977 |
| Sauces, masc sex | 0.071 | 0.072 | 0.326 | MIS | Sauces, sex | Age and study levels | All | 0.758 |
| Sauces, masc sex | 0.021 | 0.058 | 0.722 | SPMSQ | Sauces, sex | Age and study levels | All | 0.949 |
| Sauces, masc sex | 0.073 | 0.302 | 0.809 | SVF | Sauces, sex | Age and study levels | All | 0.949 |
| Tea | 0.062 | 0.03 | 0.041 | SPMSQ | Tea | Age and study levels | Masc | 0.601 |
| Tea | 0.071 | 0.046 | 0.124 | SVF | Tea | Age and study levels | Fem | 0.636 |
| Tea | 0.061 | 0.045 | 0.173 | SVF | Tea | Age, study levels and sex | All | 0.677 |
| Tea | 0.013 | 0.011 | 0.233 | MIS | Tea | Age and study levels | Fem | 0.721 |
| Tea | 0.055 | 0.045 | 0.225 | SVF | Tea | Age and study levels | All | 0.721 |
| Tea | 0.011 | 0.011 | 0.297 | MIS | Tea | Age and study levels | All | 0.74 |
| Tea | 0.011 | 0.011 | 0.307 | MIS | Tea | Age, study levels and sex | All | 0.751 |
| Tea | -0.005 | 0.009 | 0.604 | SPMSQ | Tea | Age and study levels | Fem | 0.898 |
| Tea | -0.097 | 0.195 | 0.621 | SVF | Tea | Age and study levels | Masc | 0.898 |
| Tea | -0.016 | 0.043 | 0.712 | MIS | Tea | Age and study levels | Masc | 0.949 |
| Tea | -0.001 | 0.009 | 0.9 | SPMSQ | Tea | Age, study levels and sex | All | 0.962 |
| Tea | 0 | 0.009 | 0.959 | SPMSQ | Tea | Age and study levels | All | 0.977 |
| Tea, masc sex | 0.065 | 0.037 | 0.079 | SPMSQ | Tea, sex | Age and study levels | All | 0.603 |
| Tea, masc sex | -0.165 | 0.192 | 0.392 | SVF | Tea, sex | Age and study levels | All | 0.776 |
| Tea, masc sex | -0.032 | 0.046 | 0.485 | MIS | Tea, sex | Age and study levels | All | 0.812 |
| Wine | -0.004 | 0.002 | 0.052 | SPMSQ | Wine | Age and study levels | All | 0.601 |
| Wine | -0.004 | 0.002 | 0.057 | SPMSQ | Wine | Age, study levels and sex | All | 0.601 |
| Wine | -0.004 | 0.002 | 0.102 | SPMSQ | Wine | Age and study levels | Fem | 0.612 |
| Wine | 0.016 | 0.011 | 0.156 | SVF | Wine | Age and study levels | Fem | 0.658 |
| Wine | -0.004 | 0.004 | 0.227 | SPMSQ | Wine | Age and study levels | Masc | 0.721 |
| Wine | 0.012 | 0.01 | 0.221 | SVF | Wine | Age and study levels | All | 0.721 |
| Wine | 0.012 | 0.01 | 0.233 | SVF | Wine | Age, study levels and sex | All | 0.721 |
| Wine | 0.002 | 0.005 | 0.632 | MIS | Wine | Age and study levels | Masc | 0.903 |
| Wine | -0.001 | 0.003 | 0.682 | MIS | Wine | Age and study levels | Fem | 0.937 |
| Wine | -0.004 | 0.024 | 0.875 | SVF | Wine | Age and study levels | Masc | 0.961 |
| Wine | 0 | 0.002 | 0.933 | MIS | Wine | Age and study levels | All | 0.97 |
| Wine | 0 | 0.002 | 0.937 | MIS | Wine | Age, study levels and sex | All | 0.97 |
| Wine, masc sex | -0.02 | 0.025 | 0.415 | SVF | Wine, sex | Age and study levels | All | 0.797 |
| Wine, masc sex | 0.004 | 0.006 | 0.454 | MIS | Wine, sex | Age and study levels | All | 0.801 |
| Wine, masc sex | 0 | 0.005 | 0.967 | SPMSQ | Wine, sex | Age and study levels | All | 0.977 |
